# Supplemental vitamin D enhances the recovery in peak isometric force shortly after intense exercise

**DOI:** 10.1186/1743-7075-10-69

**Published:** 2013-12-06

**Authors:** Tyler Barker, Erik D Schneider, Brian M Dixon, Vanessa T Henriksen, Lindell K Weaver

**Affiliations:** 1 The Orthopedic Specialty Hospital, 5848 S. Fashion Blvd., Murray, UT 84107, USA; 2 USANA Health Sciences, Inc, Salt Lake City, UT 84120, USA; 3 Hyperbaric Medicine, Intermountain Medical Center, Murray, UT, USA 84107 and LDS Hospital, Salt Lake City, UT 84143, USA; 4 University of Utah, School of Medicine, Salt Lake City, UT 84132, USA

**Keywords:** Vitamin D, Muscle strength, Muscle damage, Exercise

## Abstract

**Background:**

Serum 25-hydroxyvitamin D (25(OH)D) concentrations associate with skeletal muscle weakness (i.e., deficit in skeletal muscle strength) after muscular injury or damage. Although supplemental vitamin D increases serum 25(OH)D concentrations, it is unknown if supplemental vitamin D enhances strength recovery after a damaging event.

**Methods:**

Reportedly healthy and modestly active (30 minute of continuous physical activity at least 3 time/week) adult males were randomly assigned to a placebo (n = 13, age, 31(5) y; BMI, 26.9(4.2) kg/m^2^; serum 25(OH)D, 31.0(8.2) ng/mL) or vitamin D (cholecalciferol, 4000 IU; n = 15; age, 30(6) y; BMI, 27.6(6.0) kg/m^2^; serum 25(OH)D, 30.5(9.4) ng/mL) supplement. Supplements were taken daily for 35-d. After 28-d of supplementation, one randomly selected leg performed an exercise protocol (10 sets of 10 repetitive eccentric-concentric jumps on a custom horizontal plyo-press at 75% of body mass with a 20 second rest between sets) intended to induce muscle damage. During the exercise protocol, subjects were allowed to perform presses if they were unable to complete two successive jumps. Circulating chemistries (25(OH)D and alanine (ALT) and aspartate (AST) aminotransferases), single-leg peak isometric force, and muscle soreness were measured before supplementation. Circulating chemistries, single-leg peak isometric force, and muscle soreness were also measured before (immediately) and after (immediately, 1-h [blood draw only], 24-h, 48-h, 72-h, and 168-h) the damaging event.

**Results:**

Supplemental vitamin D increased serum 25(OH)D concentrations (*P* < 0.05; ≈70%) and enhanced the recovery in peak isometric force after the damaging event (*P* < 0.05; ≈8% at 24-h). Supplemental vitamin D attenuated (*P* < 0.05) the immediate and delayed (48-h, 72-h, or 168-h) increase in circulating biomarkers representative of muscle damage (ALT or AST) without ameliorating muscle soreness (*P* > 0.05).

**Conclusions:**

We conclude that supplemental vitamin D may serve as an attractive complementary approach to enhance the recovery of skeletal muscle strength following intense exercise in reportedly active adults with a sufficient vitamin D status prior to supplementation.

## Background

Vitamin D is a fat-soluble micronutrient receiving considerable clinical and research interest. These interests stem from earlier work identifying the importance of vitamin D on bone health. Since the resolution of rickets with vitamin D fortification in food, and more recently with the polarizing debates regarding the appropriate daily intake of vitamin D and the serum 25-hydroxyvitamin D (25(OH)D) concentration demarcating vitamin D sufficiency, the interest in vitamin D has spread across a range of physiological systems. Of these systems, there is mounting data indicating that vitamin D is a potent regulator of skeletal muscle physiology. In part, the regulatory interest of vitamin D on skeletal muscle physiology originated from a clinical observation [1] and diverse vitamin D interventions suggesting that increasing serum 25(OH)D concentrations could improve skeletal muscle strength or function in patients with osteomalacia [2,3].

Following these clinical findings, cell and experimental animal studies subsequently found that vitamin D regulates calcium handling and transport, phosphate metabolism, cytoskeletal protein expression, and the activation of mitogen activate protein kinase signaling pathways in skeletal muscle [4-11]. The genomic and non-genomic events established at the molecular and cellular levels extended our knowledge pertaining to the governing role of vitamin D in skeletal muscle function [for review see [12,13]. Today, the association between low serum 25(OH)D concentrations and muscle strength has evolved as results from cell and experimental animal studies are continuously translated to the causative explanation of muscular weakness in patients with low serum 25(OH)D concentrations.

The association between muscle strength and vitamin D has led to the thesis that increasing serum 25(OH)D concentrations could be a complementary approach to enhance strength (skeletal muscle) following intense exercise that induces immediate and persistent deficits in muscular strength. Consistent with this postulate, we found a positive correlation between serum 25(OH)D concentrations and strength recovery following muscular injury in subjects who were not supplementing with vitamin D [14]. Extending these findings, vitamin D treatment restored muscular strength after a crush injury in experimental rats [15]. The regenerative influence of vitamin D on skeletal muscle was attributed to the increased proliferation and decreased apoptosis of muscle cell nuclei and not to a robust alteration in satellite cell number or tissue-infiltrating leukocytes [15]. Although the results from experimental rats establish a causative influence of vitamin D on muscle regeneration after insult [15], in humans, data is less conclusive as results are limited to the association between vitamin D and strength [14,16].

The purpose of this study was, therefore, to identify the influence of supplemental vitamin D on strength recovery following intense exercise that induces a persistent (i.e., 24-h to 72-h post) deficit in peak isometric force in humans. We hypothesized that supplemental vitamin D enhances strength recovery after a damaging event. Subjects were randomly assigned to a daily placebo or vitamin D (cholecalciferol, 4000 IU) supplement in a double-blind manner. To induce persistent muscular weakness, each subject performed an intense, unilateral-leg exercise protocol consisting of repetitive eccentric-concentric contractions. In addition to monitoring single-leg muscle strength, we evaluated circulating proteins and the delayed onset of muscle soreness to assess the presence of muscle damage.

## Methods

The Urban Central Region Institutional Review Board at Intermountain Healthcare (Salt Lake City, UT, USA) approved this study. Subjects were informed of and provided written and verbal consent to the experimental protocol and procedures. Reportedly healthy and modestly-active (i.e., 30 min of continuous physically active at least 3 times per week) males were recruited to participate in this study. Potential subjects were excluding if they were taking any dietary supplements, using any prescribed or recommended medications, reported known history of any disease or condition requiring medical attention, suffered a lower leg injury during the previous year that required the use of crutches, planning on increasing or decreasing the amount of time spent in the sun or tanning bed, or traveling south of 37° N in latitude during study participation. Subjects were also excluded if they were morbidly obese (body mass index (BMI) > 40 kg/m^2^) or displayed strength or power output asymmetry (i.e., > 5% difference in peak isometric or power output between legs). Symmetry in leg strength was important since all strength testing and the exercise protocol (see below) were performed unilaterally and statistical comparisons were made within and between legs. Data was collected during the winter (between November and March) in Salt Lake City, UT, USA (40° N latitude).

### Study design and protocol

This study consisted of a randomized, double-blind, placebo-controlled experimental design. Subjects were randomly assigned to one of two groups: (1) placebo (n = 13) or (2) vitamin D (cholecalciferol, 4000 IU; n = 15) supplementation. Supplements (donated from USANA Health Sciences, Inc.) were taken daily for 35-d. During participation, subjects were asked to keep their diet consistent with their regular eating habits during the previous year and to refrain from using any dietary supplements. Subjects were also asked to refrain from physical activity, and using aspirin, ibuprofen, naproxen sodium, acetaminophen, or other anti-inflammatory agents 72-h prior to a blood draw.

Each subject provided eight fasting blood samples: (1) baseline, (Bsl and 28-d before the exercise protocol), (2) immediately before [Pre] and (3) immediately [Post], (4) 1-h, (5) 24-h, (6) 48-h, (7) 72-h, and (8) 168-h after the exercise protocol. Single-leg strength testing and perceived muscle soreness were performed on seven different occasions: (1) Bsl, (2) Pre, (3) Post, (4) 24-h, (5) 48-h, (6) 72-h, and (7) 168-h. Each visit commenced within 1-h of waking and between 06:00 and 09:00 a.m. The time remained consistent within each subject across all visits. Perceived muscle soreness was performed before the blood draw procedure, and the blood draw was performed prior to strength testing. Supplementation started at Bsl after the blood draw, perceived muscle soreness, and single-leg strength testing procedures.

### Exercise induced muscle damage

Each subject had one randomly selected leg (stretch-shortening contraction; SSC) perform an intense exercise protocol. The other leg served as the contralateral control (CON). The exercise protocol was performed 28-d after Bsl and consisted of 10 sets of 10 repetitive eccentric-concentric jumps at 75% of body mass with a 20 sec rest between each set, as described elsewhere [14,17]. Briefly, subjects were instructed and verbally encouraged to perform each set with maximal effort and to jump as-high-as possible through a full range of motion (90° of knee flexion-to-full extension). If subjects were no longer able to complete two-successive jumps, subjects were then allowed to perform presses (foot remaining on the force plate) through a full range of motion (90° of knee flexion-to-full extension). If subjects were unable to complete two successive presses, the exercise protocol was terminated. The rationale for including the presses was to allow the subjects to successfully complete the requested number of sets and repetitions. However, switching from jumps to presses presumably lowered the eccentric magnitude during loading and some subjects were unable to complete the protocol.

The mean number of jumps and presses completed during the exercise protocol were not significantly (statistically) different between the placebo (jumps, 62 ± 9; presses 19 ± 4) and vitamin D (jumps, 59 ± 8; presses, 30 ± 6) groups. Six (46%) of the placebo subjects and 9 (60%) of the vitamin D subjects completed the exercise protocol. Blood chemistries, perceived muscle soreness, and leg strength data were not significantly different between those who finished and those who did not finish the exercise protocol or between the placebo and vitamin D groups as determined by a repeated measures analysis of variance (ANOVA).

### Blood sample handling

Fasting blood samples were obtained from the antecubital vein into one 4.0 mL purple-top Becton Dickinson (BD; Franklin Lakes, NJ, USA) Vacutainer tube (K2 EDTA 7.2 mg plasma), one 4.5 mL green-top BD Vacutainer tube (PST Gel and lithium heparin, 83 units), and one 6.0 mL red-top serum BD Vacutainer tube. Plasma was separated by centrifugation (Heraeus Labofuge 400 series, Buckinghamshire, England) at 2400 *g* for 6 min within 20 min of sample collection. Following separation, plasma samples were sent to ARUP Laboratories (Salt Lake City, UT USA) for clinical chemistries (see below). After coagulation, serum was separated by centrifugation (VWR International, Clinical 50 Centrifuge) at 1100 *g* for 20 min, and then aliquoted into several small micro-centrifuge tubes. Aliquoted serum samples were stored at -80°C (Revco Freezer, GC Laboratory Equipment, Asheville, NC, USA) until analysis (see below).

### Analytical procedures

#### Serum 25(OH)D concentrations

Serum 25(OH)D concentrations (ng/mL) were measured in duplicate (coefficient of variation (CV) = 3.90%) in each blood sample, as described previously [18]. Briefly, analytes were separated on an Agilent high performance-liquid chromatography system (series 6460, Model G6460A, Santa Clara, CA, USA) and detected on an Agilent tandem mass spectrometer (Series 6410, Model G6410B, Santa Clara, CA, USA) using atmospheric pressure chemical ionization (APCI) detection (350°C gas temperature, 400°C vaporizer). The 25(OH)D_3_, deuterated 25(OH)D_3_ internal standard, and 25(OH)D_2_ precursor ions were 383.3, 386.3, and 395.4, respectively. The 25(OH)D_3_, deuterated 25(OH)D_3_, and 25(OH)D_2_ product ions were 365.3, 368.3, and 208.9, respectively. Serum 25(OH)D_2_ and 25(OH)D_3_ concentrations were determined relative to authentic standards and corrected for recovery of the 25(OH)D_3_ internal standard. Serum 25(OH)D_2_ (limit of detection = 2.0 ng/mL) was not detected in any of the subjects, and therefore, serum 25(OH)D total concentrations are referred to as serum 25(OH)D concentrations hereafter.

#### Clinical chemistries

A comprehensive metabolic panel was performed on Bsl plasma samples. Plasma AST (U/L), ALT (U/L), PTH (pg/mL), and calcium (mg/dL) concentrations were measured in each blood sample (ARUP Laboratories, Salt Lake City, UT, USA).

#### Single-leg strength testing

Single-leg strength testing was performed on a horizontal Plyo-Press (Athletic Republic, Park City, UT, USA), as described previously [17-19]. In brief, the Plyo-Press sled was adjusted for each subject to align the knee and hip joint flexion angles to 90° with the abdominal, low back region secured and stabilized to the Plyo-Press sled with a harness. Subjects performed submaximal isometric contractions on each leg at increasing intensities (≈ 50, 75, and 90%) prior to performing the testing contractions to become familiar with the testing protocol and procedure. Leg selection (i.e., CON or SSC leg) at the start of each testing session was randomized and followed by an alternating sequence of leg contractions. Peak isometric contractions (i.e., hip and knee extension) on each leg were performed in triplicate (CON leg CV = 2.62%; SSC leg CV = 6.13%). Each isometric contraction was 3 sec in duration and separated by 1 min of rest. Subjects were verbally instructed and strongly encouraged to exert maximal force against the mounted force platform. Peak isometric force was defined as the highest resultant force produced from the three isometric tests and expressed relative to body mass (N/kg).

Single-leg peak power output measures followed the single-leg isometric contractions and were measured on the same horizontal Plyo-Press with the same securing procedures described above. Starting from an extended position (full extension = 0°), subjects performed several submaximal jumps to become familiar with the testing protocol and procedure. Thereafter, subjects performed repetitive maximal effort single-leg jumps (i.e., hip and knee flexion-extension cycles). Subjects were instructed and verbally encouraged to perform the jumps as fast as possible and through a full range of motion (90° of knee flexion-to-full extension). Each test was 20 sec in duration with the weight-stack resistance set at 75% of body mass. The time-aligned product of the resultant force (N) acquired from the force platform and the weight-stack velocity (m/s) data obtained from the displacement transducer were used to calculate power output. Peak power output was defined as the highest power output produced during the 20 sec test for each leg.

Plyo-Press output data were measured from signals obtained from a mounted force plate (Advanced Mechanical Technology, Watertown, MA, USA) and from a displacement transducer (UniMeasure PA-50-NJC, Corvallis, OR, USA) attached to the weight stack. Data were sampled at 200 Hz with a low-pass filter at 10 Hz using DartPower software (Athletic Republic, Park City, UT, USA, version 2.0).

#### Muscle soreness

To assess muscular soreness, subjects lowered their body into a squat position (90° of hip and knee flexion) for 5 seconds while resting their back against a wall. This was performed in triplicate at each visit. In the squat position, subjects rated the perceived soreness of their gluteus, quadriceps, hamstrings, and calves in each leg by using a visual analog scale (10 cm in length) of 1 to 10, with 0 being ‘no pain’, 10 being the ‘worst possible pain’, and 5 being ‘tender to touch but not to contractions’ [20]. Subjects made a perpendicular mark on the visual analog scale from which perceived soreness was quantified by measuring to the nearest tenth of a centimeter.

### Statistical analyses

Data were checked for normality prior to all statistical analyses with a Shapiro-Wilk test. Statistical significance of subject characteristic and exercise protocol data (i.e., number of jump and press repetitions) were assessed using a two-sample *t*-test with a Bonferroni adjustment. Statistical significance of data (i.e., serum 25(OH)D, calcium, PTH, peak isometric force, and peak power output) were assessed using a repeated-measures ANOVA and post hoc t-tests with a Bonferroni correction on multiple pairwise comparisons when appropriate. Due to non-normally distributed data, statistical significance of ALT, AST, peak force, and perceived muscle soreness (gluteus, quadriceps, hamstrings, and calves) data were assessed with separate Friedman ANOVA tests. Statistical significance in strength recovery were assessed with a two-sample *t*-test (peak power output) with a Bonferroni correction for multiple pairwise comparisons or with a Wilcoxon Signed-Ranked Test (peak isometric force) if data were non-normally distributed. Relationships between variables were assessed with a Pearson product-moment linear correlation. All statistical analyses were performed with SYSTAT (version 13.1, Chicago, IL, USA). Statistical significance was set at *P* < 0.05. Data presented as mean (SD) unless noted otherwise.

## Results

*Subject characteristics and serum 25(OH)D concentrations*. The average serum 25(OH)D concentration was 30.8 (1.6) ng/mL upon enrollment and prior to randomization. Using the United States Endocrine Society guidelines [21], three, ten, and 15 subjects were categorized as vitamin D deficient (serum 25(OH)D < 20 ng/mL), vitamin D insufficient (serum 25(OH)D 20 to 29 ng/mL), or vitamin D sufficient (serum 25(OH)D > 30 ng/mL), respectively. Thus, for the most part, subjects displayed a good vitamin D level prior to randomization and supplementation.

Following randomization and prior to supplementation (i.e., at Bsl), subject characteristics and serum 25(OH)D concentrations were similar between the groups (Table 1). In the placebo group, six subjects were categorized as vitamin D deficient (n = 1) or insufficient (n = 5), while the majority of the subjects were vitamin D sufficient (n = 7). Likewise, several subjects were categorized as vitamin D deficient (n = 2) or insufficient (n = 5), but most subjects were vitamin D sufficient (n = 8) in the vitamin D group prior to supplementation.

**Table 1:** Baseline (Bsl) subject characteristics

	**Placebo**	**Vitamin D**
n	13	15
Age (y)	31 (5)	30 (6)
Height (cm)	180 (6)	176 (7)
Body mass (kg)	87.4 (14.0)	85.3 (17.0)
BMI (kg/m2)	26.9 (4.2)	27.6 (5.6)
25(OH)D (ng/mL)	31.0 (8.2)	30.5 (9.4)
Sodium (mmol/L)	140 (3)	142 (2)
Potassium (mmol/L)	4.28 (0.31)	4.17 (0.15)
Chloride (mmol/L)	104 (2)	105 (2)
CO2 (mmol/L)	28.0 (2.7)	27.7 (2.0)
Glucose (mg/dL)	87.1 (13.8)	86.9 (10.5)
BUN (mg/dL)	16.8 (4.1)	14.7 (3.0)
Creatinine (mg/dL)	1.00 (0.11)	1.08 (0.12)
Total protein (g/dL)	7.65 (0.33)	7.87 (0.53)
Albumin (g/dL)	4.42 (0.24)	4.64 (0.18)
Total bilirubin (mg/dL)	0.75 (0.32)	0.96 (0.48)

Data presented as mean (SD).

27 subjects were Caucasian.

1 subject was non-Hispanic black (Placebo).

**Figure 1:**
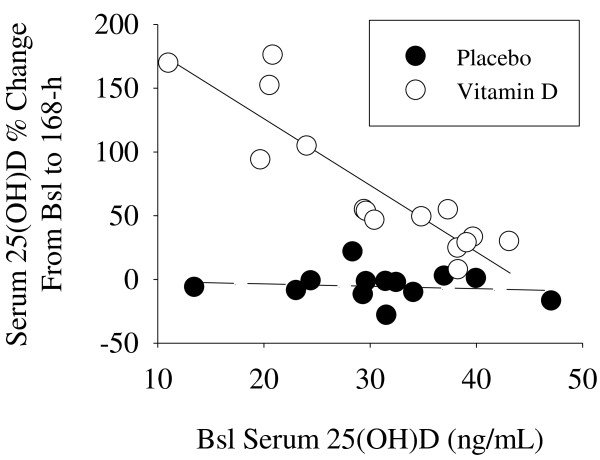
**The % change in serum 25(OH)D concentrations from Bsl to 168-h inversely correlated with Bsl serum 25(OH)D concentrations (ng/mL) in the supplemental vitamin D group (r = -0.90, *P*** **< 0.05, solid linear regression line) but not in the placebo group (r = -0.14, *P*** **= 0.66, dashed linear regression line).**

At the conclusion of supplementation (i.e., 168-h [or 35-d total of supplementation]), the number of vitamin D deficient, insufficient, and sufficient subjects remained constant in the placebo group compared to that at Bsl. Likewise, the serum 25(OH)D concentration did not significantly change in the placebo group from Bsl to 168-h (29.1 (7.9) ng/mL) (Additional file 1: Figure S1). Conversely, serum 25(OH)D concentrations significantly increased (≈ 70%, range = 7 – 175%) in the vitamin D group at 168-h (47.9 (7.6) ng/mL). The % increase in serum 25(OH)D concentrations from Bsl to 168-h inversely correlated with Bsl serum 25(OH)D concentrations in the supplemental vitamin D group (Figure 1), which confirms and extends those reported previously [22].

**Figure 2:**
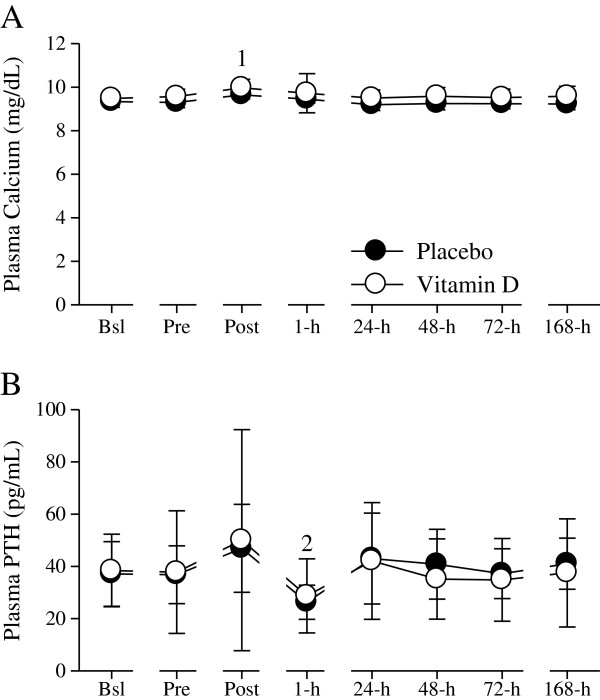
**Plasma calcium and PTH concentrations. (A)** Plasma calcium concentrations (mg/dL) were significantly (^1^*P* < 0.05 vs. Bsl, Pre, 1-h, 24-h, 48-h, 72-h, and 168-h) increased at Post. **(B)** Plasma PTH concentrations (pg/mL) were significantly (^2^*P* < 0.05 vs. Pre, Post, 72-h, and 168-h) decreased at 1-h. Figure legend provided in ‘A’. Data presented as mean (SD).

*Damaging-exercise modulates circulating calcium and PTH concentrations*. The increase in plasma calcium (≈ 4%) concentrations at Post and the decrease (≈ 26%) in PTH concentrations at 1-h (Figure 2) were not modulated by supplemental vitamin D. Although supplemental vitamin D did not significantly moderate calcium or PTH changes, there were, however, fluctuations in calcium and PTH. Plasma calcium concentrations increased above the clinical reference range (8.2 to 10.4 mg/dL) in four-supplemental vitamin D subjects after the exercise protocol (at Post, 1-h, 48-h, or 168-h). Plasma PTH concentrations increased above the reference range (15 to 75 pg/mL) in one-placebo subject (at 24-h) and in two-supplemental vitamin D subjects (at Pre, Post, 24-h, 72-h, or 168-h). One subject in the supplemental vitamin D group displayed a plasma PTH concentration below the clinical reference range at 1-h.

**Figure 3:**
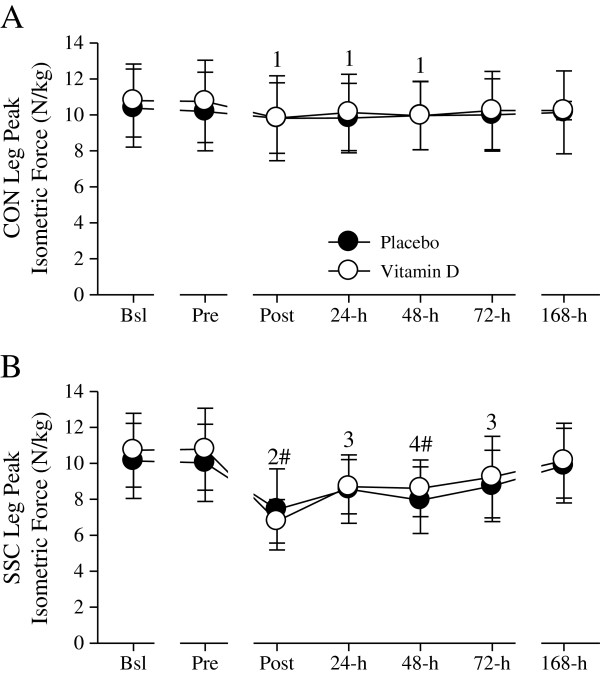
**Single-leg peak isometric force (N/kg).** CON- **(A)** and SSC- **(B)** leg peak isometric forces were not significantly different between the placebo and supplemental vitamin D groups. Peak isometric force significantly decreased in the CON leg at Post, 24-h, and 48-h (^1^*P* < 0.05 vs. Bsl and Pre). In the SSC leg, peak isometric force was significantly decreased at Post (^2^*P* < 0.05 vs. Bsl, Pre, 24-h, 48-h, 72-h, 168-h), 24-h (^3^*P* < 0.05 vs. Bsl, Pre, and 168-h), 48-h (^4^*P* < 0.05 vs. Bsl, Pre, 72-h, and 168-h), and 72-h (^3^*P* < 0.05 vs. Bsl, Pre, and 168-h). Peak isometric force was significantly (^#^*P* < 0.05) different between the CON and SSC legs at Post and 48-h. Figure legend provided in ‘A’. Data presented as mean (SD).

*Single-leg peak isometric force and peak power output*. As illustrated in Figure 3, peak isometric force was decreased (≈ 6%) in the CON leg immediately and the first 48-h after the damaging event. The peak isometric force deficits in the SSC leg, however, were more severe (≈ 32% at Post, 17% at 24-h, 21% at 48-h, and 14% at 72 h) and persisted for a longer duration than those in the CON leg. Compared to the CON leg, peak isometric force was significantly decreased in the SSC leg at Post (≈ 28%) and 48-h (≈ 17%). Peak isometric force in the CON and SSC legs were not significantly different between the placebo and vitamin D groups.

**Figure 4:**
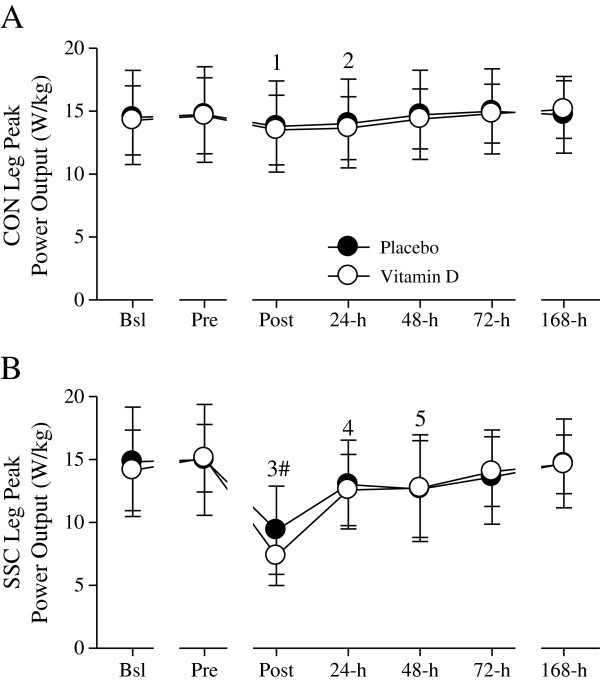
**Single-leg peak power output (W/kg).** CON- **(A)** and SSC- **(B)** leg peak power outputs were not significantly different between the placebo and supplemental vitamin D groups. Peak power output was significantly decreased at Post (^1^*P* < 0.05 vs. Pre, 24-h, 48-h, and 72-h) and 24-h (^2^*P* < 0.05 vs. 48-h and 72-h) in the CON leg. In the SSC leg, peak power output was significantly decreased at Post (^3^*P* < 0.05 vs. Bsl, Pre, 24-h, 48-h, 72-h, and 168-h), 24-h (^4^*P* < 0.05 vs. Bsl, Pre, 72-h, and 168-h), and 48-h (^5^*P* < 0.05 vs. Pre and 168-h). Peak power output was significantly (^#^*P* < 0.05) different between the CON and SSC legs at Post. Figure legend provided in ‘A’. Data presented as mean (SD).

Similar to peak isometric force, the peak power output-deficits in the SSC leg were more pronounced (≈ 43% at Post, 12% at 24-h, and 12% at 48-h) and persisted for a longer duration than those in the CON leg (≈ 4 and 5% at Post and 24-h, respectively; Figure 4). Peak power output in the SSC leg was significantly decreased (≈ 39%) compared to the CON leg at Post. Peak power outputs in the CON and SSC leg were not significantly different between the placebo and vitamin D groups.

**Figure 5:**
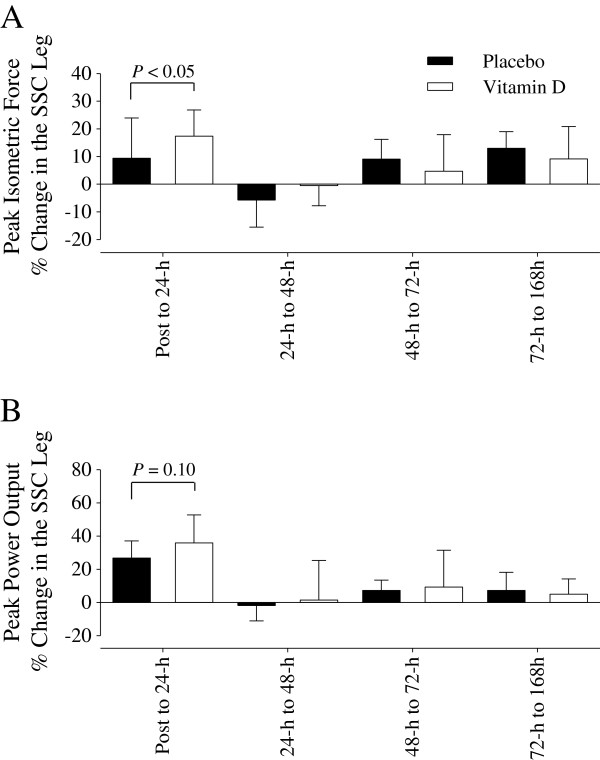
**The recovery (%) in peak isometric force and peak power output in the SSC leg. (A)** The peak isometric force % change from Post to 24-h was significantly (*P* < 0.05) increased in the supplemental vitamin D group compared to that in the placebo group. **(B)** The peak power output % change from Post to 24-h tended (*P* = 0.10) to be increased in the supplemental vitamin D group compared to that in the placebo group. Figure legend provided in ‘A’. Data presented as mean (SD).

A planned comparison prior to data collection was to normalize the deficits in the SSC leg to the CON leg. Considering the decrements in the CON leg, however, this comparison would underestimate the deficit in the SSC leg induced by the exercise protocol. Therefore, we examined the recovery in peak isometric force and peak power output in the SSC leg and between groups at each time interval after the exercise protocol. The recovery of peak isometric force from Post to 24-h in the SSC leg was significantly greater (≈ 8%) in the supplemental vitamin D group (Figure 5A). There was a similar trend (*P* = 0.10) for a greater (≈ 9%) recovery of peak power output in SSC leg from Post to 24-h in the supplemental vitamin D group as well (Figure 5B).

**Figure 6:**
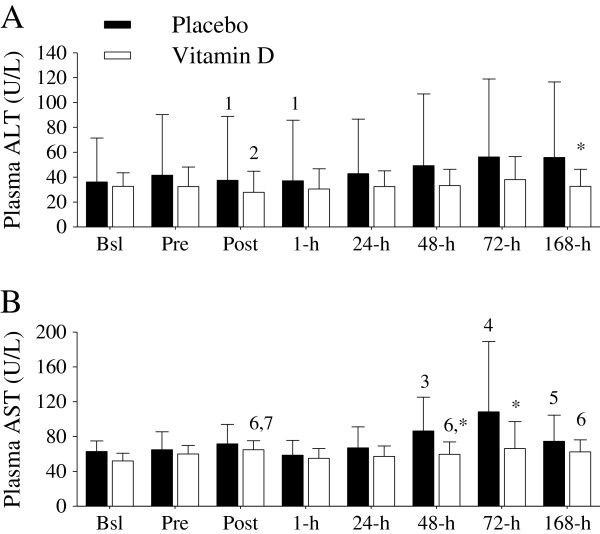
**Plasma ALT (U/L) and AST (U/L). (A)** Plasma ALT increased (^1^*P* < 0.05 vs. 24-h, 48-h, 72-h, and 168-h) in the placebo group days after the exercise protocol compared to those at Post and 1-h. Plasma ALT was significantly (^2^*P* < 0.05 vs. Bsl, Pre, 24-h, 48-h, and 72-h) decreased at Post in the supplemental vitamin D group. Supplemental vitamin D significantly (*P < 0.05) decreased ALT compared to the placebo group at 168-h. **(B)** Plasma AST significantly (^3^*P* < 0.05, 48-h vs. Bsl and 1-h; ^4^*P* < 0.05, 72-h vs. Bsl, Pre, 1-hr, and 24-hr; ^5^*P* < 0.05, 168-h vs. 1-h) increased in the placebo group during the days following the exercise protocol. In the supplemental vitamin D group, plasma AST was significantly increased at Post (^6^*P* < 0.05, vs. Bsl; ^7^*P* < 0.05 vs. 1-h), 48-h (^6^*P* < 0.05, vs. Bsl), and 168-h (^6^*P* < 0.05, vs. Bsl). Supplemental vitamin D significantly (^*^P < 0.05) attenuated the AST increase at 48-h and 72-h compared to those in the placebo group. Figure legend provided in ‘A’. Data presented as mean (SD).

*Circulating biomarkers of muscle damage*. The persistent deficits in peak isometric force and peak power output after the exercise protocol provide evidence of skeletal muscle damage [23,24]. Skeletal muscle damage is also identified by increases in blood proteins, such as ALT and AST [23,25]. In this investigation, supplemental vitamin D blunted the ALT and AST increases after (i.e., at 48-h, 72-h, and 168-h) the damaging event (Figure 6A and B).

**Figure 7:**
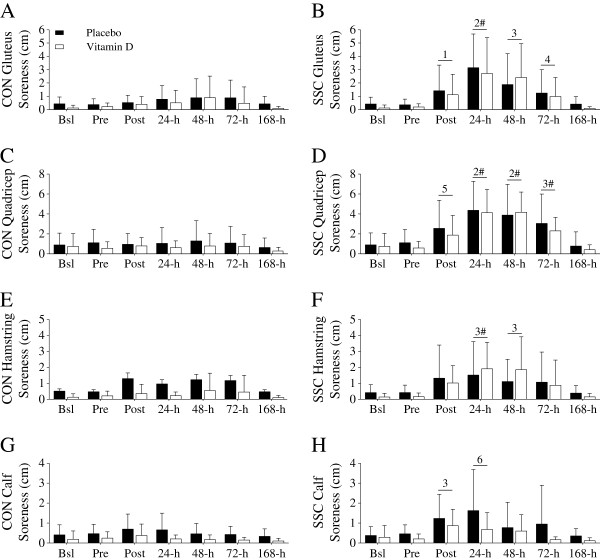
**Perceived muscle soreness (cm).** Perceived soreness in the gluteus, quadriceps, hamstrings, or calves were not significantly different between the placebo and supplemental vitamin D groups. **(A-B)** Gluteus soreness increased in the SSC leg at Post (^1^*P* < 0.05 vs. 24-h and 168-h), 24-h (^2^*P* < 0.05 vs. Bsl, Pre, 72-h, and 168-h), 48-h (^3^*P* < 0.05 vs. Bsl, Pre, and 168-h), and 72-h (^4^*P* < 0.05 vs. 168-h). Gluteus soreness was significantly (^#^*P* < 0.05) different between the CON and SSC leg at 24-h. **(C-D)** Quadriceps soreness increased in the SSC leg at Post (^5^*P* < 0.05 vs. Pre, 24-h, 48-h, and 168-h), 24-h (^2^*P* < 0.05 vs. Bsl, Pre, 72-h, and 168-h), 48-h (^2^*P* < 0.05 vs. Bsl, Pre, 72-h, and 168-h), and 72-h (^3^*P* < 0.05 vs. Bsl, Pre, and 168-h). Quadriceps soreness was significantly (^#^*P* < 0.05) different between the CON and SSC leg at 24-h, 48-h, and 72-h. **(E-F)** Hamstring soreness increased at 24-h (^3^*P* < 0.05 vs. Bsl, Pre, and 168-h) and 48-h (^3^*P* < 0.05 vs. Bsl, Pre, and 168-h). Hamstring soreness was significantly (^#^*P* < 0.05) different between the CON and SSC leg at 24-h. **(G-H)** Calf soreness increased at Post (^3^*P* < 0.05 vs. Bsl, Pre, and 168-h) and 24-h (^6^*P* < 0.05 vs. 72-h and 168-h). Calf soreness was not significantly different between the CON and SSC legs. Figure legend provided in ‘A’. Data presented as mean (SD).

*Delayed onset of muscle soreness*. Muscle soreness increased immediately and persisted for 24-h to 72-h after the damaging event in the SSC leg (Figure 7A-H). Supplemental vitamin D was ineffective at abrogating muscle soreness in the SSC leg.

## Discussion

The intense exercise protocol induced muscular weakness that persisted for several days (2- to 3-d), which is indicative of skeletal muscle damage [for review see [23]. The recovery in peak isometric force shortly after the intense exercise protocol was enhanced with supplemental vitamin D. Supplemental vitamin D also attenuated the increase in circulating biomarkers representative of muscle damage after the exercise protocol. These novel findings provide evidence that supplemental vitamin D may serve as an attractive complementary approach to enhance strength recovery shortly after unaccustomed or repetitive contractions that induce persistent muscular weakness in humans.

The enhanced recovery in peak isometric force shortly after (i.e., Post to 24-h) intense exercise suggests that vitamin D is modulating mechanisms of muscle weakness. A compelling study conducted in severely vitamin D-deficient (i.e., serum 25(OH)D < 6.0 ng/mL (or 15 nmol/L)) subjects illustrated recently that vitamin D treatment (20,000 IU on alternate days for 10-12 weeks) increased serum 25(OH)D concentrations and reduced the phosphocreatine recovery half-time [26]. Thus, in theory, vitamin D could enhance strength recovery after intense exercise by altering phosphate metabolism or accumulation in skeletal muscle [4,27]. Despite the faster short-term recovery, vitamin D did not enhance the delayed (i.e., 24- to 72-h) recovery in peak isometric force after the intense exercise protocol. Therefore, it is plausible that vitamin D is modulating mechanisms of muscle weakness-induced by fatigue as opposed to muscle damage.

Among other measurement tools, muscle damage is commonly determined by an increase in circulating creatine kinase (CK) or a persistent deficit in skeletal muscle force production [23,24]. In experimental rats, vitamin D attenuated the increase in plasma CK activity following intense exercise training [28] and improved force production following a crush injury [15]. Therefore, vitamin D treatment attenuated the increase in systemic and local biomarkers of muscle damage in experimental rats.

In addition to augmenting strength recovery, supplemental vitamin D ameliorated the increase in plasma ALT and AST after the exercise protocol. The circulating measurements of ALT and AST are used clinically to diagnosis liver damage or failure. ALT and AST are also found in skeletal muscle and released into the circulation after acute muscular damage [29,30]. Thus, supplemental vitamin D could abrogate muscle damage. Instead of inhibiting release from skeletal muscle into the circulation, however, it is plausible that supplemental vitamin D is modulating ALT and AST through an alternative pathway. Clearly future research is needed to confirm the findings from this study, but it is noteworthy that supplemental vitamin D blunted the increase in circulating enzymes representative of skeletal muscle damage after enhancing strength recovery.

Although it is beyond the scope of the present investigation, there are several mechanisms that could explain the enhanced recovery in peak isometric force with supplemental vitamin D. First, mitochondrial oxidative phosphorylation reportedly increases with vitamin D treatment in severely vitamin D-deficient adults [26], which in theory could enhance recovery. Second, supplemental vitamin D could enhance strength recovery by inhibiting apoptosis and increasing extracellular matrix proteins [15]. Third, supplemental vitamin D increases serum 25(OH)D concentrations, which consequentially, could increase the vitamin D receptor (VDR) in skeletal muscle [28,31]. The increased expression of the VDR could impact muscle regeneration and function as it regulates protein synthesis [32] and calcium transport [6]. However, the existence of the VDR in skeletal muscle is controversial [33] and future research is needed to confirm the influence of increasing serum 25(OH)D concentrations on the expression of the VDR in skeletal muscle. Finally, CYP27B1 increases in regenerating skeletal muscle [11]. The increase in CYP27B1 could increase the conversion of 25(OH)D to 1,25(OH)D, which is the predominately-active metabolite form of vitamin D that could aid in muscle regeneration. Thus, there are several putative mechanisms that vitamin D could modulate to enhance strength recovery after intense exercise.

Supplemental vitamin D at 4000 IU/d does not modulate plasma calcium [34] but significantly decreases plasma PTH [35] in adults with initially low serum 25(OH)D concentrations (i.e., < 20 ng/mL). In this study consisting of subjects with mostly sufficient serum 25(OH)D concentrations prior to supplementation, supplemental vitamin D did not significantly influence plasma calcium or PTH concentrations. It is noteworthy, however, that one subject displayed a decrease in PTH below its reference range and four subjects displayed increases in plasma calcium above its reference range after the exercise protocol in supplemental vitamin D group. Since these observations were not found prior to exercise, it is likely that the decrease in PTH and increase in calcium were mediated by physical exertion as opposed to supplemental vitamin D. Conversely, PTH concentrations increased above its reference range before and after the exercise protocol in two-supplemental vitamin D subjects. The PTH increases occurred in the absence of concomitant fluctuations in plasma calcium above or below its reference range. Therefore, the physiological impact of PTH increases following daily supplemental vitamin D at 4000 IU is unclear. Given the inter-regulatory actions [36-39], future research addressing the influence of supplemental vitamin D on PTH and calcium prior to and following intense exercise is warranted. Future studies are also encouraged to consider possible plasma volume, magnesium level, and ionized calcium changes when investigating the role of vitamin D on PTH fluctuations prior to and after intense exercise.

There are a couple of limitations of the exercise protocol that should be discussed. First, some subjects performed the exercise protocol until volitional exhaustion while others completed the protocol. Thus, there are likely differences in exercise volume between subjects. Along these lines, there was a tendency for the vitamin D group to perform less jumps but more presses, although these differences were not significant between groups. Second, the vitamin D group tended to display greater decreases in peak isometric forces and peak power outputs at Post. This could suggest greater fatigue after the exercise protocol and a greater capacity for a faster recovery. Future studies are needed to confirm the present findings and would benefit from a controlled exercise volume.

As alluded to above, a limitation to this study is that it does not identify if supplemental vitamin D is modulating fatigue- or damage-related mechanisms, or both, to enhance strength recovery after the exercise protocol. Considering that vitamin D enhanced recovery the first 24-h and not thereafter suggests that vitamin D might be altering fatigue as opposed to damage-related mechanisms of weakness. Additionally, muscle biopsies were not obtained to ascertain microscopic alterations in skeletal muscle indicative of damage. Another limitation of this study is that subjects were not block randomized according to vitamin D status prior to supplementation. Also, this study was conducted in a modest number of reportedly healthy and active subjects, who for the most part, were vitamin D sufficient prior to supplementation. We speculate that the good vitamin D levels are the result of healthy lifestyles and dietary habits since none of the subjects were supplementing.

## Conclusions

Muscular weakness induced by intense or unaccustomed movements is a major impairment that hinders millions of people worldwide every year. We provide new data demonstrating that supplemental vitamin D could enhance the recovery of force production after intense exercise that mediates a persistent deficit in muscular strength. Supplemental vitamin D also attenuated the increase in circulating biomarkers representative of muscle damage. These findings were observed in subjects with good vitamin D levels, for the most part, and who were modestly active and reportedly healthy prior to supplementation. Based on these findings, we conclude that supplemental vitamin D may serve as an attractive complementary approach to enhance the recovery of skeletal muscle strength following intense exercise in subjects who are vitamin D sufficient and physically active prior to supplementation.

## Abbreviations

ALT: Alanine aminotransferase; AST: Aspartate aminotransferase; CON: Control leg; PTH: Parathyroid hormone; SSC: Exercise leg; 25(OH)D: 25-hydroxyvitamin D.

## Competing interests

ES and BD are employed by USANA Health Sciences, Inc.

## Author contributions

TB, BD, and LW contributed to the conception and design of the experiments. TB, ES, BD, VH, and LW contributed to the collection, analysis, and/or interpretation of the data. TB drafted the article, and TB, ES, BD, VH, and LW revised it critically for important intellectual content. All the authors approved the final version of the manuscript and qualify for authorship, and all of those who qualify for authorship are listed.

## Supplementary Material

Additional file 1: Figure S1 Serum 25(OH)D concentrations (ng/mL). Serum 25(OH)D concentrations increased in the placebo (^1^*P* < 0.05 vs. Bsl, Pre, 1-h, 48-h, 72-h, and 168-h) and supplemental vitamin D (^2^*P* < 0.05 vs. Pre and 72-h) groups at Post. Supplemental vitamin D increased serum 25(OH)D concentrations (^3^*P* < 0.05 vs. Bsl; *P < 0.05 vs. corresponding Placebo). Data presented as mean (SD). Click here for file
