# Supplementary material for: Supplemental vitamin D enhances the recovery in peak isometric force shortly after intense exercise
Source: Nutr Metab (Lond). 2013 Dec 6;10:69. doi: 10.1186/1743-7075-10-69 (PMC4029611; doi:10.1186/1743-7075-10-69)
Supplement: Additional file 1: Figure S1 — Serum 25(OH)D concentrations (ng/mL). Serum 25(OH)D concentrations increased in the placebo (1P < 0.05 vs. Bsl, Pre, 1-h, 48-h, 72-h, and 168-h) and supplemental vitamin D (2P < 0.05 vs. Pre and 72-h) groups at Post. Supplemental vitamin D increased serum 25(OH)D concentrations (3P < 0.05 vs. Bsl; *P < 0.05 vs. corresponding Placebo). Data presented as mean (SD). [file 1743-7075-10-69-S1.docx]

**Additional file 1: Figure S1.** Serum 25(OH)D concentrations (ng/mL). Serum 25(OH)D concentrations increased in the placebo (^1^*P* < 0.05 vs. Bsl, Pre, 1-h, 48-h, 72-h, and 168-h) and supplemental vitamin D (^2^*P* < 0.05 vs. Pre and 72-h) groups at Post. Supplemental vitamin D increased serum 25(OH)D concentrations (^3^*P* < 0.05 vs. Bsl; **P* < 0.05 vs. corresponding Placebo). Data presented as mean (SD).
